# Statin use reduces cardiovascular events and all-cause mortality amongst Chinese patients with type 2 diabetes mellitus: a 5-year cohort study

**DOI:** 10.1186/s12872-017-0599-x

**Published:** 2017-06-24

**Authors:** Colman Siu Cheung Fung, Eric Yuk Fai Wan, Anca Ka Chun Chan, Cindy Lo Kuen Lam

**Affiliations:** Department of Family Medicine and Primary Care, The University of Hong Kong, 3/F Ap Lei Chau Clinic, 161 Main Street, Ap Lei Chau, Hong Kong, Hong Kong, Special Administrative Region of China

**Keywords:** Statin, Cardiovascular events, All-cause mortality, Chinese, Type 2 diabetes mellitus, Cohort study

## Abstract

**Background:**

The benefit of statin on the management of Type 2 Diabetes Mellitus (T2DM) among Chinese patients in primary care is not clear nor fully implemented in clinical practice. This study aimed to evaluate and quantify the benefit of statin on the overall cardiovascular risk and all-cause mortality in patients with T2DM.

**Methods:**

Uncomplicated diabetic patients with baseline low-density-lipoprotein cholesterol (LDL-C) > 2.6 mmol/L and without statin use before baseline in 2010 were followed-up for 5 years for cardiovascular disease (CVD) events and all-cause mortality. Propensity score matching analysis was conducted to identify patients who were newly prescribed statin at baseline and then compared to non-statin users with similar baseline characteristics. Subgroup analysis was done within the statin group to detect any difference in outcomes between patients achieving target LDL-C of <2.6 mmol/L and not. Multivariable Cox proportional hazards regression with adjustment of all baseline covariates was used to evaluate the effect of statin on outcome events. Hazard ratio (HR) and its 95% confidence intervals were reported.

**Results:**

10,104 pairs of diabetic patients were propensity score matched. Statin users had an extra drop of 1.21 mmol/L in LDL-C than non-users. Statin group had a CVD incidence rate of 16.533 per 1000 person-years whereas comparison group had 32.387 per 1000 person-years (HR: 0.458) during a median follow-up period of 50.5 months. Statin group had a mortality rate of 8.138 deaths per 1000 person-years whereas comparison group had 19.603 deaths per 1000 person-years (HR: 0.378). For patients prescribed with statin, the HR was 0.491 for CVD and 0.487 for all-cause mortality if target of LDL-C < 2.6 mmol/L achieved compare to those not achieved.

**Conclusions:**

Use of statin was associated with a significant decrease in CVD risk and all-cause mortality among diabetic patients in primary care, and the risk reduction was most significant if the target of LDL-C less than 2.6 mmol/L was achieved.

## Background

The global trend of aiming at reduction of the overall cardiovascular risk in the management of diabetic patients makes the lipid-lowering class of drugs-statin one of the commonly prescribed medications in the drug regimen of patients diagnosed with Type 2 Diabetes Mellitus (T2DM). Statin, with its main pharmacological effect being lowering of the low density-lipoprotein cholesterol (LDL-C) level, also has the potential to lower triglyceride and raise high-density-lipoprotein cholesterol, and hence it was commonly used as the first-line oral medication to lower lipid level and reduce the associated cardiovascular risk. A LDL-C target of 2.6 mmol/L or below is commonly set for patients with T2DM. Many guidelines or practitioners view T2DM as a cardiovascular event equivalent, and suggest LDL-C level should be lowered to below 1.8 mmol/L. Although lifestyle intervention including diet and regular physical activity are advocated for patients to control their hyperlipidemia, the dramatic effect of statin on the drop of LDL-C and the comparable few side-effects make statin a popular option in the management of patients with T2DM. Many Western studies had affirmed the benefit of statin on the lipid-lowering effect and the reduction of cardiovascular events. Meta-analysis showed that amongst patients without established cardiovascular diseases but with cardiovascular risk factors, statin use was associated with significant improvement in survival and large reductions in major cardiovascular events risks [1]. However, the impact of statin on the overall management of T2DM in Asian populations, including Chinese population is not clear. Little evidence can be found on how the use of statin can lower the cardiovascular risk and all-cause mortality. This gap is imminently needed as the coverage of statin in diabetic patients is lower comparable to Western population. Chinese patients are reluctant to use statin and one of the reasons is the fear of the potential side-effect of liver impairment. Many Chinese patients have an excuse that they can eat more healthily and do more exercise, so that they do not need to start using lipid-lowering agent. However, what is observed is that they cannot reach the lifestyle modification level that may have the equivalent impact on improving their lipid profile by lipid-lowering agent. Solidify the benefits of lipid-lowering agent can give an option to the diabetic patients and their doctors on how the lipid profile can be improved. This study aimed to evaluate the benefit of statin on the overall cardiovascular risk and all-cause mortality in patients with T2DM, so that physicians could have more evidence to discuss with their patients regarding the use of statin in the management plan of T2DM. The objectives of the study were to reveal the hazard ratios of cardiovascular diseases and all-cause mortality by comparing patients with T2DM taking statin versus those not taking statin in the primary care setting. This comparison was particularly meaningful in primary care settings because uncomplicated diabetic patients were encouraged to be taken care by primary care physicians while diabetic patients with complications may need to be taken care by secondary or tertiary care providers. If statin use could be shown being able to prevent significantly CVD or related complications at primary care level, the need of secondary or tertiary healthcare services can be greatly reduced, thus saving the associated expensive medical cost at secondary or tertiary care level. In addition, the health-related quality of life of the diabetic patients can also be maintained as they have their complications prevented or delayed.

## Methods

### Study design

This retrospective cohort study included Chinese patients with T2DM having their diabetes being followed-up in public primary healthcare. The dataset was retrieved from an evaluation of a large-scale local evaluation of diabetes care [2]. Data were gathered through the administratives from the central computerized database under the Hospital Authority (HA), the largest government organization in-charge of all public hospitals and out-patient clinics in Hong Kong, between 1 January 2010 and 31 December 2010.

Adult Chinese (aged 18 years or above) bearing a clinical diagnosis of T2DM, without prior CVD or liver disease history, and having follow-up in primary care clinics of HA between 1 January 2010 and 31 December 2010 were included in the study. The clinical diagnosis of T2DM was identified using the International Classification of Primary Care-2 (ICPC-2) code of ‘T90’ through the administrative database of HA and the CVD identification was described in the subsequent section. The baselines for statin and comparison groups were defined as the date of first prescription with statin and the first attendance record in primary care clinics for DM follow-up, respectively. Patients with new prescription of statin at baseline were classified as statin group, and those with prior statin prescription record before baseline, or with LDL-C < 2.6 mmol/L at baseline, or stopped statin within 1 year after baseline were excluded. Patients not undertaking statin at or before baseline were classified as comparison group, and were excluded if they had any statin treatment within 1 year after baseline. Since there was no standardized definition or protocol of lifestyle changes in the care plan of diabetic patients, all diabetic patients in both groups received lifestyle modification advice and education upon their T2DM follow-up consultation as usual.

### Exposures

Follow-up started at baseline and continued until a switching to or addition of another anti-diabetic medication, the date of incidence of the first outcome event, all-cause mortality, a censoring event or last contact with any in-patient and out-patient services of HA as censoring until the end of study on 30 November 2015. Generally, the interval between follow-up consultations for diabetic patients in HA general out-patient clinics was from three to four months, and the supply of the chronic medications (including statin) matches with the follow-up interval. The prescription seldom exceeds a maximum of 120 days’ drug supply. Patients with T2DM who were not put on any chronic medications may have a longer follow-up period but would not be more than six months (180 days) usually. Thus, the censoring events were 121 days of no drug prescription record for statin group and 181 days of no attendance record in out-patient clinics for comparison group.

### Outcomes: Cardiovascular events and mortality

The outcomes of interest were events with one of the following subtype diagnoses: 1) CVD event with one of the following diagnoses: coronary heart disease (CHD), stroke, or heart failure, 2) CHD, 3) stroke, 4) heart failure and 5) all-cause mortality. The diagnosis of comorbidities was identified by the diagnosis coding system of ICPC-2 and International Classification of Diseases, Ninth Edition, Clinical Modification (ICD-9-CM).

The earliest date of diagnosis with ICPC-2 of K74 to K76 or ICD-9-CM of 410.x, 411.x to 414.x, 798.x was marked as the time of CHD (including ischaemic heart disease, myocardial infarction, coronary death) or sudden death event. The earliest date of diagnosis with ICPC-2 of K77 or ICD-9-CM of 428.x was the time of heart failure event. The earliest date of diagnosis with ICPC-2 of K89 to K91 or ICD-9-CM of 430.x to 438.x was the time of stroke event (including fatal and non-fatal). All-cause mortality was determined using the Hong Kong Death Registry population data.

### Baseline covariates

Baseline covariates of patients were categorized into four branches: [1] socio-demographics, [2] clinical parameters, disease characteristics and treatment modalities. Socio-demographics of patients covered age, gender and smoking status. Clinical parameters consisted of haemoglobin A1c (HbA1c), systolic and diastolic blood pressure (SBP & DBP), lipid profile [LDL-C and total cholesterol to high-density lipoprotein-cholesterol ratio (TC/HDL-C) and triglyceride (TG)] and body mass index (BMI). Disease characteristics encompassed self-reported T2DM duration, presence of chronic kidney disease (indicated by baseline estimated glomerular filtration rate (eGFR) < 60 ml/min/1.73m^2^), Charlson’s comorbidity index, hypertension (defined by the clinical diagnosis with ICPC-2 code of “K86” or “K87”) and family history of DM. Treatment modalities included the usages of insulin, oral diabetic and anti-hypertensive drugs. All laboratory assays were performed in accredited HA laboratories by the College of American Pathologists, the Hong Kong Accreditation Service or the National Association of Testing Authorities, Australia.

### Data analysis

Patients with similar baseline characteristics were selected from statin and comparison groups by means of propensity score matching (PSM) analysis. Propensity score modelled the probability of statin using multivariable logistic regression with the adjustment of all baseline covariates. The propensity score mapping was made using a one-to-one matching with the nearest neighbour, within 0.001 caliper and without replacement approach.

Baseline characteristics of demographics, clinical parameters, disease characteristics and treatment modalities were summarized using descriptive statistics. Differences in baseline characteristics between statin and comparison groups were tested using independent t-tests for continuous variables or chi-square tests for categorical variables. The pre- and post- changes in clinical parameters (HbA1c, SBP, DBP, LDL-C, TC/HDL-C ratio, TG and BMI) for statin and comparison groups were examined using paired t-tests separately and the difference in difference between statin and comparison groups was evaluated using independent t-tests.

The CVD incidence rate was estimated by an exact 95% confidence interval (CI) based on a Poisson distribution [3]. Kaplan-Meier survival curves were reported and log-rank test compared the differences in survival rates between groups. Multivariable Cox proportional hazards regression with the adjustment of all baseline covariates was used to evaluate the effect of statin group comparing with comparison group on the outcome events. Hazard ratio (HR) and its 95% CI was reported for each variable within the regression models. The proportional hazards assumption was assessed by examining plots of the scaled Schoenfeld residuals against time for the predictors. Presence of multi-collinearity was checked by examining the variance inflation factor. Eventually, a sensitivity analysis was performed by excluding subjects with follow-up period less than two years.

All significance tests were two-tailed. A *p*-value <0.05 was considered statistically significant. All statistical analyses were performed using STATA Version 13.0.

## Results

**Fig. 1:**
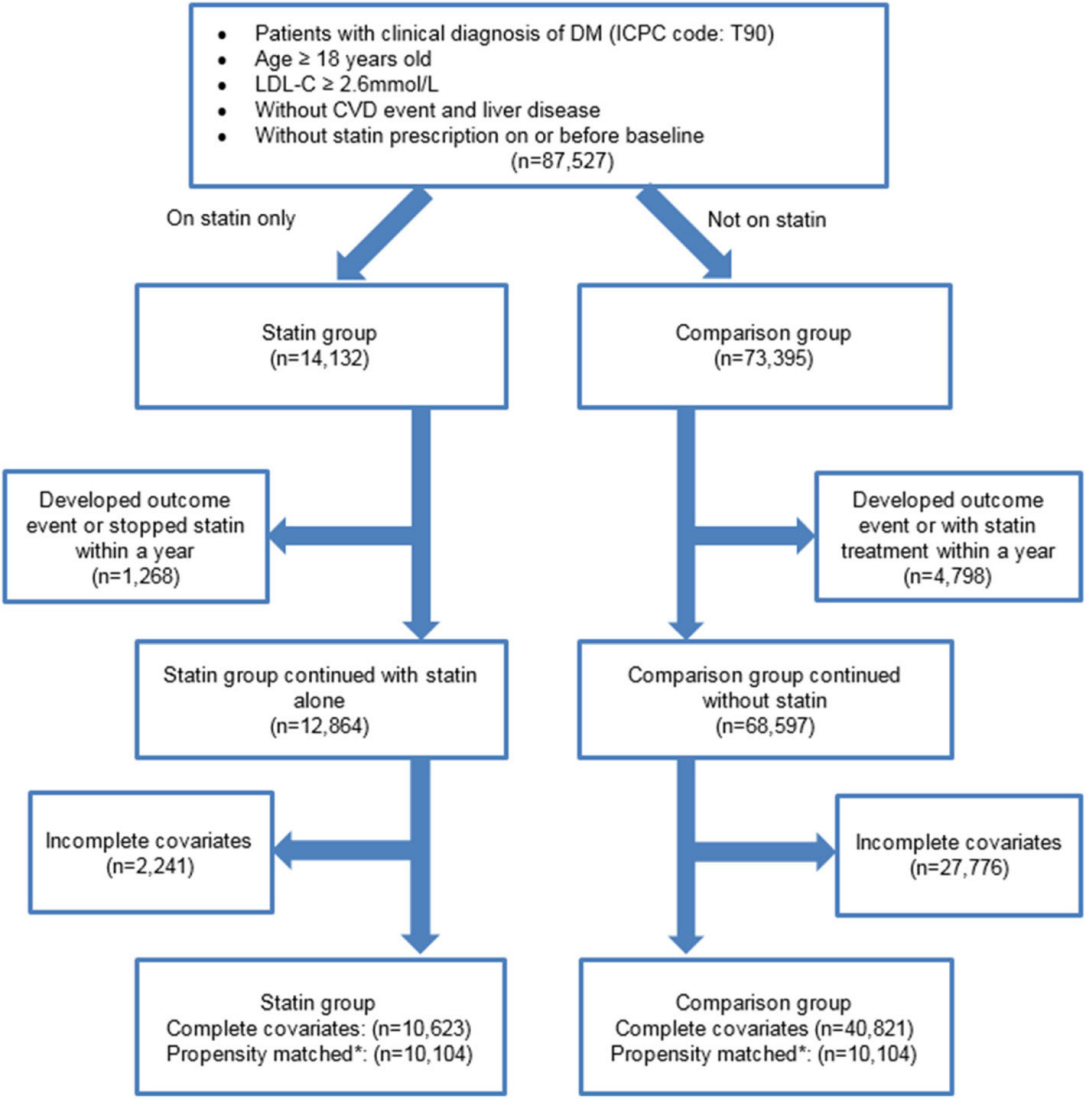
Flow chart of subjects matching and comparison. *LDL-C* Low Density Lipoprotein Cholesterol; *CVD* Cardiovascular Disease. * Matched subjects with propensity score matching using factors including age, gender, smoking status, HbA1c, BP, LDL-C, TC/HDL-C ratio, triglyceride, BMI, presence of chronic kidney disease, duration of DM, hypertension, family history of DM, use of anti-hypertensive drugs use of lipid lowering agents and Charlson Index

Figure 1 summarizes the flow of subjects in the study. Originally, there were 14,132 (16.1%) and 73,395 (83.9%) eligible subjects in statin and comparison groups under the primary care of HA between 1 January 2010 and 31 December 2010, respectively. After the exclusion of subjects having management changed within one year from baseline and missing data of baseline covariates, remaining subjects in statin and comparison groups were 10,623 and 40,821, respectively. 10,104 subjects from each group were successfully matched with each other using propensity score algorithm, regarding to all baseline characteristics.

Table 1 displays the baseline characteristics between the two groups after matching. The two groups showed no significant difference in terms of all baseline characteristics after PSM. Table 2 compares the key clinical parameters between baseline and post-programme for both statin and comparison groups. All clinical parameters showed an improvement for both groups. As indicated by the difference in difference, statin group had significant improvements in SBP, DBP, LDL-C, TC/HDL-C ratio and triglyceride than the comparison group after the programme.

**Table 1:** Baseline characteristics between statin and comparison groups

Factor	Statin Group (*N* = 10,104)	Comparison Group (*N* = 10,104)	*P*-value
Socio-Demographic (%, *n*)			
Age (Mean ± SD, *n*), year	64.80 ± 10.32 (10,104)	64.82 ± 11.19 (10,104)	0.889
Gender			0.743
Female	58.19% (5880)	58.42% (5903)	
Male	41.81% (4224)	41.58% (4201)	
Smoking Status			0.796
Non-smoker	89.96% (9090)	90.07% (9101)	
Smoker	10.04% (1014)	9.93% (1003)	
Clinical Parameters (Mean ± SD)			
HbA1c, %	7.31 ± 1.28 (10,104)	7.30 ± 1.33 (10,104)	0.505
SBP, mmHg	134.98 ± 16.51 (10,104)	134.85 ± 16.88 (10,104)	0.580
DBP, mmHg	74.94 ± 9.92 (10,104)	74.91 ± 10.05 (10,104)	0.865
LDL-C, mmol/L	3.76 ± 0.63 (10,104)	3.75 ± 0.64 (10,104)	0.615
BMI, kg/m^2^	25.73 ± 3.88 (10,104)	25.79 ± 3.91 (10,104)	0.289
TC/ HDL-C Ratio	4.76 ± 1.11 (10,104)	4.76 ± 1.09 (10,104)	0.997
Triglyceride, mmol/L	1.61 ± 0.79 (10,104)	1.62 ± 0.86 (10,104)	0.600
Disease Characteristics (%, *n*)			
Duration of DM (Mean ± SD), years	7.11 ± 6.38 (10,104)	7.10 ± 6.25 (10,104)	0.881
Presence of Chronic Kidney Disease (%, *n*)	4.13% (417)	3.96% (400)	0.544
Charlson’s Index (Mean ± SD)	3.95 ± 1.05 (10,104)	3.96 ± 1.12 (10,104)	0.496
Hypertension	75.81% (7660)	75.93% (7672)	0.844
Family History of DM	46.61% (4709)	47.01% (4750)	0.563
Treatment Modalities (%, *n*)			
Use of Insulin	1.39% (140)	1.44% (145)	0.765
Use of Oral Diabetic Drugs	88.08% (8900)	87.91% (8882)	0.697
Use of Anti-Hypertensive Drugs	78.66% (7948)	79.05% (7987)	0.502

*HbA1c* Haemoglobin A1c**,** *SBP* Systolic Blood Pressure**,** *DBP* Diastolic Blood Pressure**,** *LDL-C* Low Density Lipoprotein - Cholesterol**,** *BMI* Body Mass Index**,** *TC* Total Cholesterol**,** *HDL-C* High Density Lipoprotein - Cholesterol**,** *DM* Diabetes Mellitus

*Significant with *p*-value <0.05 by chi-square test or t-test as appropriate

**Table 2:** Clinical parameter comparisons between baseline and post

Clinical Parameters (Mean ± SD)	Statin Group Paired Diff.^a^ (*N* = 10,104)	Comparison Group Paired Diff.^a^ (*N* = 10,104)	Diff. in diff. (Statin Group - Comparison Group)	*P*-value
HbA1c, %	−0.18 ± 1.31	−0.21 ± 1.29	0.02	0.223
SBP, mmHg	−5.31 ± 19.81	−3.28 ± 19.20	−2.03	< 0.001*
DBP, mmHg	−4.06 ± 10.89	−1.96 ± 10.49	−2.09	< 0.001*
LDL-C, mmol/L	−1.47 ± 0.79	−0.26 ± 0.64	−1.21	< 0.001*
TC/ HDL-C Ratio	−1.39 ± 0.99	−0.40 ± 1.04	−1.00	< 0.001*
Triglyceride, mmol/L	−0.25 ± 0.75	−0.12 ± 0.79	−0.14	< 0.001*
BMI, kg/m^2^	−0.25 ± 1.81	−0.26 ± 1.62	0.01	0.699

*HbA1c* Haemoglobin A1c**,** *SBP* Systolic Blood Pressure**,** *DBP* Diastolic Blood Pressure**,** *LDL-C* Low Density Lipoprotein - Cholesterol**,** *TC* Total Cholesterol**,** *HDL-C* High Density Lipoprotein - Cholesterol**,** *BMI* Body Mass Index

*Significant with *p*-value <0.05 by independent t-test

^a^Paired difference (Post - baseline)

Table 3 shows the number and incidence rates of all-cause mortality and CVD events. Statin group had lower incidence rates in all-cause mortality and CVD events than the comparison group. During a median follow-up period of 50.5 months, statin group had a CVD incidence rate of 16.533 per 1000 person-years whereas comparison group had 32.387 per 1000 person-years (Number needed to treat (NNT) = 54). Similarly, statin group had an incidence rate of 8.138 deaths per 1000 person-years whereas comparison group had 19.603 deaths per 1000 person-years (NNT = 51). Further breakdown of the CVD events into CHD, stroke and heart failure also showed consistent results as that of the all-cause mortality and CVD events.

**Table 3:** Number and incidence rates of CVD event and all-cause mortality

Event	Cumulative Incidence		Incidence Rate (Cases/ 1000 Person-years)		Person-years
	No. of Event	Rate	Estimate	95% CI*	
Statin Group (*N* = 10,104)					
CVD	789	7.81%	16.533	(15.400, 17.728)	47,722.2
CHD	382	3.78%	7.902	(7.129, 8.736)	48,341.8
Stroke	312	3.09%	6.458	(5.761, 7.216)	48,313.9
Heart Failure	196	1.94%	4.036	(3.491, 4.643)	48,560.5
All-cause Mortality	397	3.93%	8.138	(7.357, 8.980)	48,781.3
Comparison Group (*N* = 10,104)					
CVD	977	9.67%	32.387	(30.387, 34.483)	30,166.8
CHD	476	4.71%	15.691	(14.313, 17.166)	30,336.1
Stroke	410	4.06%	13.534	(12.256, 14.910)	30,293.0
Heart Failure	240	2.38%	7.913	(6.943, 8.980)	30,331.3
All-cause Mortality	596	5.90%	19.603	(18.061, 21.242)	30,402.8

*CVD* Cardiovascular Disease, *CHD* Coronary Heart Disease, *DM* Diabetes Mellitus, *CI* Confidence Interval

*The 95% CI was constructed based on Poisson distribution

Multivariable Cox proportional hazard regressions were performed on the dependent variables of all-cause mortality and CVD event and results are tabulated in Table 4. The variance inflation factors ranged from 1 to 5.08, which indicated no violation of proportional hazard and absence of multi-collinearity. Observed random scattered points from the scaled Schoenfeld residual plots indicated the proportional hazard assumption was satisfied. There was a 62.2% risk reduction of all-cause mortality in statin group compared to comparison group and the difference in their survival time was highly significant (*P* < 0.001) by log-rank test. Moreover, in terms of the incidence of CVD events (including CHD, stroke and heart failure), patients in statin group had around 55–60% risk reduction when compared to the comparison group. Log-rank test also suggested that there were significant differences (*P* < 0.001) in the survival time of all CVD events between the two groups. Sensitivity analysis was conducted with the exclusion of subjects whose follow-up periods were less than two years and similar results were obtained.

**Table 4:** Multivariable Cox proportional hazard regression on the dependent variables of CVD event and all-cause mortality

	Statin Group Comparing with Comparison Group		
	HR^a^	95%CI	*P*-value
Main Analysis (*N* = 20,208)			
CVD	0.458	(0.415,0.504)	<0.001*
CHD	0.436	(0.380,0.502)	<0.001*
Heart Failure	0.488	(0.402,0.593)	<0.001*
Stroke	0.429	(0.368,0.499)	<0.001*
All-cause Mortality	0.378	(0.331,0.431)	<0.001*
Sensitivity Analysis (*N* = 15,776)			
CVD	0.522	(0.462,0.589)	<0.001*
CHD	0.467	(0.390,0.559)	<0.001*
Heart Failure	0.581	(0.454,0.742)	<0.001*
Stroke	0.516	(0.428,0.622)	<0.001*
All-cause Mortality	0.382	(0.325,0.450)	<0.001*

Sensitivity analysis: exclude subjects with follow-up period less than 2 years

*CVD* Cardiovascular Disease, *CHD* Coronary Heart Disease, *HR* Hazard Ratio, *CI* Confidence Interval

*p-value <0.05

^a^Hazard ratios were adjusted by age, gender, smoking status, haemoglobin A1c, systolic blood pressure, diastolic blood pressure, triglyceride, low density liporotein-cholesterol, body mass index, total cholesterol-to-high density lipoprotein-cholesterol ratio, presence of chronic kidney disease, duration of diabetes mellitus, Charlson Index, diagnosed hypertension, family history of diabetes mellitus and usages of insulin, oral and anti-hypertensive drugs index at baseline

Sub-group analysis was performed on patients started with statin to differentiate patients achieving target of LDL-C < 2.6 mmol/L and those who did not (Table 5). Diabetic patients who were put on statin had HR of 0.444 to 0.553 for CVD and all-cause mortality if they achieved the target of LDL-C < 2.6 mmol/L.

**Table 5:** Multivariable Cox proportional hazard regression on the dependent variables of CVD event and all-cause mortality within statin group

	Post LDL-C < 2.6 mmol/L (*n* = 7641) comparing with post LDL-C ≥ 2.6 mmol/L (*n* = 2463)		
	HR^a^	95%CI	*P*-value
Main Analysis (*N* = 10,104)			
CVD	0.491	(0.422,0.571)	<0.001*
CHD	0.477	(0.385,0.591)	<0.001*
Heart Failure	0.444	(0.329,0.600)	<0.001*
Stroke	0.553	(0.433,0.708)	<0.001*
All-cause Mortality	0.487	(0.393,0.602)	<0.001*

*LDL-C* Low Density Lipoprotein-Cholesterol, *CVD* Cardiovascular Disease, *CHD* Coronary Heart Disease

*p-value <0.05

^a^Hazard ratios were adjusted by age, gender, smoking status, haemoglobin A1c, systolic blood pressure, diastolic blood pressure, triglyceride, low density liporotein-cholesterol, body mass index, total cholesterol-to-high density lipoprotein-cholesterol ratio, presence of chronic kidney disease, duration of diabetes mellitus, Charlson Index, diagnosed hypertension, family history of diabetes mellitus and usages of insulin, oral and anti-hypertensive drugs index at baseline

## Discussion

This study is by far the latest analysis on a large population scale to quantify the beneficial impact of using statin on diabetic patients under the primary care, by comparing directly the different clinical parameters and cardiovascular complications and all-cause mortality. Our study was coherent with other Western trials that greater LDL-C reduction led to greater CV risk reduction [4, 5]. Statin is well known for lowering LDL-C. This study manifested that patients with T2DM who were prescribed with statin for their associated hyperlipidemia had significant lipid-lowering effects (in terms of decreasing LDL-C, TC/HDL-C, and TG) in Chinese diabetic population. Our study confirmed that further to lipid-lowering effect, use of statin itself was associated with risk reduction of suffering from various CVD complications and all-cause mortality. Statin approximately halved the CVD risk, and reduced all-cause mortality by more than 60%. Our sub-group analysis showed that if patients undertook statin and had achieved the target of LDL-C < 2.6 mmol/L at the same time, the risk reduction was much greater. Apart from its lipid-lowering effect, statin may reduce the complications through other mechanisms like ameliorating endothelial function,, keeping plaque stability, abating thrombus formation, and modulating inflammatory responses, etc. [6]. The pleiotropic effects of statins on reducing CVD events included amelioration of endothelial dysfunction, normalized vasomotion, enhanced nitric oxide bioavailability, antioxidant effects, anti-inflammatory effects, plaque stabilization, stimulation of endothelial progenitor cell recruitment, immunomodulation, and inhibition of myocardial hypertrophy [7]. The beneficial pleiotropic effect of statin could account for the CV risk drop even in those patients prescribed with statin but did not achieve the LDL-C target. However, the risk reduction effect of statin is more prominent if the LDL-C can be kept to an optimal level. This leads to the discussion of adding other newer lipid-lowering agents which act on different mechanisms like ezetimibe or PCSK9 Inhibitors [8]. Statin is advocated and used as the first-line lipid-lowering medication globally for hyperlipidemia (particularly hypercholesterolaemia) unless contraindicated. One challenging clinical decision family physicians or general practitioners have to make is to decide if and when a diabetic patient with hyperlipidemia should start prescribing statin, particularly at the early stage of management of their diabetes in order to reduce the overall cardiovascular risk and complications. Findings from this study provide more evidence and help family physicians or general practitioners to make a shared decision with their diabetic patients.

At baseline, the number of diabetic patients with hyperlipidemia who were not taking statin was 5 times of those taking statin. This was similar to other studies which showed the inadequate use of statin and the sub-optimal control of LDL-C [9, 10]. For example, less than half of the US adults with elevated LDL-C levels received treatment to lower their cholesterol, and less than 33% of patients achieved their target LDL-C levels despite using currently available treatments [10]. It was common for Chinese diabetic patients to keep observing their elevated LDL-C instead of starting statin. They had the health belief that LDL-C level would go down if they eat more healthily and do some more exercise, which they usually over-estimate their ability to comply with the required level of life style modifications. On the other hand, some physicians may reinforce this “non-pharmacological” approach of management, hoping to motivate their patients more adhere to healthy lifestyle modifications through diet regulations and regular exercise pattern. Patients’ reluctance to use lipid-lowering drugs mostly originated from the underlying fear of the potential harmful effects on their liver, and the need to take the lipid-lowering drugs life-long [11]. Drug compliance would also be an issue because most of them experienced no hyperlipidemia-related symptoms, and they did not have objective measurements of their lipid level frequently like that of the home blood glucose or home blood pressure monitoring. They would also like to try alternative ways including herbs to lower their lipid-level [11, 12]. Some patients may turn to alternative medicine or Chinese medicine practitioners, which is not uncommon in Chinese community as traditional Chinese medicine has its own school of theory and management on hyperlipidemia [12]. A recent review suggested that the concern of side-effects of statin, which was still an underlying reason of the under-use of statin amongst patients with high CVD risk like diabetic patients, may be exaggerated, and that an informed decision on statin use for CVD events prevention should be advocated [13]. The financial influence on the statin prescription in our study population was negligible as the consultation fee was HKD 45 [USD 5.78] only under public primary care without additional drug fee for statin prescription.

While the concept of lifestyle modification is rather abstract, more and more studies are now suggesting that the association between diet and hyperlipidemia may not be that strong [9]. Some patients who claim to have very low level of fat intake in their diet are still found to have hyperlipidemia. There are some other possible factors like genetic or comorbidities having a stronger association with hyperlipidemia, and hence diet control alone may not be helpful. More studies pointed that the circulating cholesterol was indeed manufactured by liver rather than absorbed through diet [14]. Blocking the hepatic synthesis of cholesterol may have a more promising outcome of reducing LDL-C and subsequent CVD risk and mortality than diet modification.

The magnitude of LDL-C reduction from our study was comparable to studies from other regions [7], suggesting that statin worked well on Chinese patients in reducing LDL-C. Comparison group had shorter median follow-up period (30.5 months) than the statin group (63.5 months) which suggested that comparison group had a higher probability of having changes in their condition (either the control of hyperlipidemia or an event occurrence) that warrants a change of their management of hyperlipidemia. There was one local prospective cohort study in 1996 to 2005 on lipid control and use of lipid-lowering medications for prevention of cardiovascular events in Chinese T2DM patients [15]. It showed that statins use was associated with lower CVD risk with a HR 0.66 (0.50–0.88). There were several potential reasons why our HR (0.458 to 0.522) was lower than that. Our study sample was larger, with more female patients, fewer smokers, lower systolic BP, lower baseline HbA1c, and higher baseline LDL-C (more room for LDL-C reduction). All these can contribute to the lower HR in our study than the previous study.

After controlling the severity of hyperlipidemia (as indicated by LDL-C) and other factors, a patient on statin had 62.2% and 54.2% lower risk of having all-cause mortality and any of the CVD event when compared to a patient not on statin. Such risk reductions may be partially attributed by the concurrent reduction in SBP, DBP, TC/HDL-C, and TG. With reference to the United Kingdom prospective diabetes study (UKPDS) and a meta-analysis from Cholesterol Treatment Trialists’ (CTT) Collaboration in which every 1 mmol/L reduction in LDL-C was associated with risk reduction of CVD by 22%, stroke by 21%, CHD by 12% and all-cause mortality by 9% [16, 17], our study revealed that diabetic patients with statin use may well have a risk reduction in CVD, stroke, CHD and all-cause mortality by 54%, 57%, 56% and 62%, respectively, when compared to statin non-users.

The latest 2016 ESC/EAS Guidelines for the Management of dyslipidemias recommends patients with high CV risk (including diabetic patients), an LDL-C goal of <2.6 mmol/L, or a reduction by at least 50% if the baseline LDL-C is between 2.6 and 5.2 mmol/L [18]. The comparison of our matched cohort showed that the statin group had a 39% reduction while the non-statin comparison group had only 6.9% reduction in LDL-C. Further studies are needed to take into account the dosage of the statin to see if the newly recommended 50% reduction of LDL-C is applicable to Asian population, and comparing those having at least 50% reduction in LDL-C to those who cannot on the subsequent actual reduction in CVD complications or all-cause mortality. However, our current finding of a 39% reduction in LDL-C level in diabetic patients using statin is still encouraging.

With reference to the overall significant beneficial potential of statin in the management of hyperlipidemia and reduction in the cardiovascular risk and all-cause mortality in diabetic patients, family doctors or general practitioners should discuss with their patients in details about the role of lipid-lowering medications in their long-term management plan. In the interim, the role of lifestyle modifications, including healthy diet, regular exercise, and weight control, are equally important as patients with chronic diseases are now encouraged towards being more self-empowered and self-enabled [19]. Nonetheless, any statin-related side-effects such as liver impairment or allergic reaction should be alerted.

### Strengths and limitations of the study

The study sample size was comparatively large. Comparison was done on propensity-score matched subjects to eliminate any influence from other confounding factors like age, severity of hyperlipidemia and diabetes mellitus at baseline, etc. Sensitivity analysis was performed in addition to full analysis to try to reduce any potential bias. As most findings were consistent from both full cohort with complete case and propensity score-matched cohort analytical approaches, stain was strongly suggested to be beneficial to diabetic patients with hyperlipidemia regarding their clinical parameters and outcomes.

There are a few weaknesses in our study. Firstly, five years may not be long enough for some cases of cardiovascular complications or mortality to develop. A longer period of follow-up can help to confirm if long-term use of statin is still associated with lower cardiovascular risk or all-cause mortality. Secondly, details on the use of statin including the exact dosage of statin taken by each individual patient, were not available. Drug compliance to statin could not be assessed in our study. However, we performed the within group analysis by comparing patients achieved LDL-C target with those did not within the statin group. We assumed that those patients prescribed with statin and achieved LDL-C target would have good drug compliance while poor drug compliance may be one of the reasons for patients prescribed with statin but the LDL-C target was not achieved. The drawbacks of using statin, including side-effects or intolerance had not been taken into account for analysis. Also, the extent of lifestyle modifications of the patients had not been recorded and thus could not be taken into consideration of its effect on the improvement in the lipid profile and the subsequent cardiovascular risk reduction.

## Conclusions

Hyperlipidemia with LDL-C ≥ 2.6 mmol/L was prevalent in diabetic patients. Statin was still under prescribed. Use of statin was associated with a significant decrease in cardiovascular disease risk and all-cause mortality in diabetic patients, and the risk reduction was more outstanding if the statin could help the diabetic patients to achieve their target LDL-C of less than 2.6 mmol/L.

## Abbreviations

BMI: Body Mass Index
CHD: Coronary Heart Disease
CI: Confidence Interval
CTT: Cholesterol Treatment Trialists’
CVD: Cardiovascular Diseases
DBP: Diastolic Blood Pressure
DM: Diabetes Mellitus
eGFR: Estimated Glomerular Filtration Rate
HA: Hospital Authority
HbA1c: Haemoglobin A1c
HR: Hazard Ratio
ICD-9-CM: International Classification of Diseases, Ninth Edition, Clinical Modification
ICPC-2: International Classification of Primary Care-2
IRB: Institutional Review Board
LDL-C: Low-Density Lipoprotein-Cholesterol
NNT: Number Needed to Treat
SBP: Systolic Blood Pressure
T2DM: Type 2 Diabetes Mellitus
TC/HDL-C ratio: High-Density-Lipoprotein Cholesterol Ratio
TG: Triglyceride
UKPDS: United Kingdom Prospective Diabetes Study
